# Rehabilitation professionals’ perspectives and experiences with the use of technologies for violence prevention: a qualitative study

**DOI:** 10.1186/s12913-023-09789-7

**Published:** 2023-08-23

**Authors:** Alisa Grigorovich, Pia Kontos, Milos R. Popovic

**Affiliations:** 1 Recreation and Leisure Studies, Brock University, St Catharines, Canada; 2 KITE Research Institute, Toronto Rehabilitation Institute – University Health Network, Toronto, Canada; 3 Dalla Lana School of Public Health, University of Toronto, Toronto, Canada; 4 Institute of Biomedical Engineering, University of Toronto, Toronto, ON Canada

**Keywords:** Electronic flagging, Cameras, Alarms, Occupational health, Patient safety

## Abstract

**Background:**

There is growing public policy and research interest in the development and use of various technologies for managing violence in healthcare settings to protect the health and well-being of patients and workers. However, little research exists on the impact of technologies on violence prevention, and in particular in the context of rehabilitation settings. Our study addresses this gap by exploring the perceptions and experiences of rehabilitation professionals regarding how technologies are used (or not) for violence prevention, and their perceptions regarding their efficacy and impact.

**Methods:**

This was a descriptive qualitative study with 10 diverse professionals (e.g., physical therapy, occupational therapy, recreation therapy, nursing) who worked across inpatient and outpatient settings in one rehabilitation hospital. Data collection consisted of semi-structured interviews with all participants. A conventional approach to content analysis was used to identify key themes.

**Results:**

We found that participants used three types of technologies for violence prevention: an electronic patient flagging system, fixed and portable emergency alarms, and cameras. All of these were perceived by participants as being largely ineffective for violence prevention due to poor design features, malfunction, limited resources, and incompatibility with the culture of care. Our analysis further suggests that professionals’ perception that these technologies would not prevent violence may be linked to their focus on individual patients, with a corresponding lack of attention to structural factors, including the culture of care and the organizational and physical environment.

**Conclusions:**

Our findings suggest an urgent need for greater consideration of structural factors in efforts to develop effective interventions for violence prevention in rehabilitation settings, including the design and implementation of new technologies.

## Background

Incidents of patient-to-patient and patient-to-health professional violence are an underreported, ubiquitous, and persistent public health problem across care settings. In Ontario Canada alone, the health/community care sector has one of the highest rates of occupational injuries due to violence as compared to other sectors [1, 2]. Violence can be physical (e.g. hitting, grabbing, biting) and verbal (e.g. ridicule, threats), and can lead to serious injuries and psychological repercussions for both patients and workers; it is associated with increased burnout, job dissatisfaction, harm to self and others, disruption to care, and decreased feelings of safety [1–4].

There is growing public policy and research interest in the development and use of various technologies for managing violence in healthcare settings including electronic patient flagging systems, video cameras, bed and door sensors, and personal alarms [2, 5–9]. The main anticipated benefits of using these are that they are thought to improve prevention through earlier or more accurate identification of incidents (and their precipitating factors) and improved response time. However, little research exists on their impact on violence prevention in healthcare settings, and no research to date has focused on their use within rehabilitation settings. Existing interventions for violence prevention are largely based on those developed for emergency and other acute care environments, and thus it is unclear whether these are applicable to rehabilitation settings. Rehabilitation hospitals are unique in that they encompass a multi-disciplinary workforce of allied health professionals that work in interprofessional teams to support individuals with complex medical, physical, and emotional needs. For example, in Ontario, rehabilitation hospitals generally include physiotherapy, occupational therapy, speech-language pathology, recreation therapy, social work and nursing services that are provided to people with injury, chronic condition, or disability (e.g. neurological, musculoskeletal, psychiatric, cardiac). To the best of our knowledge, the perceptions and experiences of rehabilitation professionals regarding technologies for violence prevention have yet to be investigated. Given that research on health technologies more broadly has identified that professionals’ lack of trust, familiarity, and/or acceptance of technologies is a key barrier to implementation and uptake [10–12], research with rehabilitation professionals is critical to understand their desire and need for technologies and the barriers to their implementation and use. Our interest was precisely to address this significant gap in knowledge and understanding about how technologies are used (or not) for violence prevention in rehabilitation settings, and the perceptions of rehabilitation professionals regarding their efficacy and impact.

## Methods

### Design

We conducted a descriptive qualitative study using semi-structured interviews.

### Setting

An academic hospital specializing in adult rehabilitation and complex continuing care in an urban setting in Ontario, Canada. This setting includes out-patient rehabilitation services for individuals living with an injury, disability, or chronic illness (e.g., brain injury, dementia, spinal cord injury, musculoskeletal conditions) as well as inpatient rehabilitation for a short (< 3 months) or longer term for individuals in need of 24/7 support given their complex conditions. In this setting, technologies are part of broader organizational health and safety programs that encompass violence prevention, including policies, annual risk assessments, incident reporting systems, education, and training for professionals on ways to manage violent behaviours and to summon immediate assistance, and ongoing statistical review of violence related events (e.g., incidents, near misses). Given how little research exists on the use of technology for violence prevention in rehabilitation settings, we chose to focus on this aspect of these programs.

### Recruitment and data collection

Participants were recruited using a study flyer that was distributed via email by managers to rehabilitation professionals across the study setting. The flyer included details about the study aims and methods, and contact information. Ten participants were recruited for the study representing diverse rehabilitation professions (e.g., physical therapy, occupational therapy, recreation therapy, and nursing). All but one was a woman, and most worked in inpatient unit settings with persons living with neurological conditions (e.g., brain injury, dementia). Data collection took place between November 2019 and April 2020 and consisted of 1–1 semi-structured interviews. All interviews were conducted either in person or over the phone by the first author (AG) and lasted approximately 60 min. An interview guide with open-ended questions was used (See Table 1 for examples of questions asked). All the interviews were audio recorded with permission of the participants and were professionally transcribed. To ensure the confidentiality of the study participants, identifying details of respondents were subsequently removed from the transcripts. To ensure methodological rigour and trustworthiness a written record was maintained of all audio recordings, transcripts, interview guides, and data analysis processes and products, including field notes and coding memos [13, 14].

**Table 1:** Examples of interview questions

• *Describe what training/education you have received around aggression and violence and its prevention in rehabilitation.* o *How useful do you find these to be for this purpose?* o *What would make these more useful/effective for this purpose?*
• *Describe the strategies and policies you have in your workplace around managing aggression and preventing violence* o *How useful do you find these to be for this purpose?* o *What would make these more useful/effective for this purpose?*
• *What technologies have you observed or know about that are used for violence prevention in your workplace?* o *How useful do you find these to be for this purpose?* o *What would make these more useful/effective for this purpose?* o *What training or education have you received about these?*
• *Can you describe a specific example of using technology for managing/responding to aggression or violence in your workplace?*
• *What policies/guidelines exist around the use of technologies for violence prevention in your workplace?*

### Data analysis

We followed a conventional approach to content analysis, which is an approach that adheres to the naturalistic paradigm and is used to understand a phenomenon when existing theory or the research literature regarding the phenomenon is limited [15]. The approach is one that is data-driven (i.e., the researcher stays close to the text) and thus does not require the development of preconceived categories or theoretical perspectives. Instead analysis is guided primarily by the research question and study aim, pertinent assumptions, or general ‘sentisizing concepts’ [16] that provide a starting point for the development of initial analytical categories and guide interpretation. Specifically, our analysis was informed by sensitizing concepts from sociological research on violence and on the adoption and use of technologies in health care settings [1, 17–21]. Examples of concepts we used include agency and structure, context, culture of care, professional roles and care practices, normalization of violence, alarm fatigue, reductionism, resistance, and compliance. Analysis was primarily inductive and iterative and began with the first and second authors reading the transcripts several times to obtain an overall understanding of the data in their entirety. In the next step, each author independently performed an initial line-by-line coding of participants’ answers and then created categories and summarized these into major themes (e.g., the usefulness of technology for violence prevention) and minor themes (e.g., mistrust) for each open-ended question. The two authors then met to discuss these preliminary themes and together developed the final set of codes that the first author then used to sort and label all the data in NVivo 12. After all of the data were labeled, the authors then met again to review and further analyse the abstracted and labelled data for underlying meaning and content and to further develop the major themes. Any disagreements were discussed until consensus was reached.

### Ethical considerations

Ethical approval for this study was granted by the University Health Network Research Ethics Board. All participants received written and verbal information about the study prior to the interview and all provided written informed consent to participate in the interviews and for the data to be used in presentations and publications. To ensure the anonymity of the participants, interview excerpts are identified by the type of rehabilitation unit rather than by profession.

## Findings

We have organized our analysis of the data into one overarching theme – *Technology won’t prevent violence* - and 3 sub-themes including *behavioural safety alerts, alarms, and cameras*, which collectively capture rehabilitation professionals’ perceptions that the technologies in their institution are ineffective for violence prevention and the reasons why they feel this way.

### Technology won’t prevent violence

#### Behavioral safety alerts

The most common form of technology implemented for violence prevention across all units was an electronic flagging system called the Behavioral Safety Alert [BSA], which is an alert that is created in the electronic patient record (EPR) to indicate that a patient (or an associated visitor) poses a risk of violence based on the fact that they have previously displayed violent behaviour. This was implemented at the hospital for all units following a recent change to the Occupational Health and Safety Act of Ontario (1990) that mandates that all employers and supervisors must communicate to employees about “a person with a history of violent behaviour” or “the risk of violent, aggressive or responsive behaviour by patients, residents or clients in the workplace” if they are expected to encounter these individuals in their work and if their risk of workplace violence is likely to expose workers to physical injury. The purpose of doing so is to ensure that they understand the risks associated with the person and to understand what triggers may lead to violence so that they may be avoided. While all the participants were aware of this technology and its purpose of violence prevention, most chose not to use it when interacting with their patients. Its lack of prominence or visibility in the EPR was one reason cited by participants, as noted in the following:

So those [BSAs] are entered into the computer system, into EPR, and then a little flag goes up on the electronic white board to indicate one’s there, and then it’s supposed to identify kind of the behaviour triggers, [and] strategies to mitigate. But to be honest, I don’t find that they really pop out at you. You know, I know patients who have had BSAs entered before, and I use the computer system all the time and just don’t really notice them. (102, Complex Continuing Care)

Another participant explained that BSAs are not very useful because professionals often were already aware that a particular patient could potentially be violent. They stated that they would know about this from colleagues, or because they expected that most individuals that they cared for, given their condition, would be flagged for violence. For example, when asked whether this was something she reviewed before interacting with a new patient, this participant replied:

To be honest, because I’m someone who…works with…inpatients…really after they’ve arrived, I will have already known [them]. We’re not the biggest group, we’re not a huge hospital. So, for example, if I get a referral, an inpatient referral, someone’s probably already told me about it before I actually get the referral. And usually that person will be like, “Oh they’re also really pleasant” or…just some random tidbit of information. So, if they had a behavioural concern, they will tell me. They might say, “Oh by the way, he also had a brain injury” that’s the type of side information I’m getting. So, I haven’t found the need to go and check the chart vigorously for that kind of stuff, because…[it’s] already been flagged probably by the referring therapist. (108, Brain & Spinal Cord)

Similarly, another respondent reported: "I think it’s not very effective, because…all the patients [here on our unit] have a behaviour safety alert…we’re all aware of their behaviours, so we’re not going to go into the behaviour safety alerts and read what it is." (109, Specialized Dementia Unit)

BSAs sometimes included mention of “contributing factors,” if these were known, and a care management plan that was intended to help professionals to avoid triggering an incident by modifying their approach to care or scheduling of activities. As an example of this, a participant described a care plan for a patient who is “prone to punching out when given a bed bath” as including the following recommendations: “try to do the bed bath in pairs with two nurses and watching out for the right arm. Or you know, wait until family comes to do the bath, so that they can help to calm the patient, like things like that.” (102, Complex Continuing Care). However, not all patients with BSAs had care plans that were this specific or even included explicit strategies that could be used to avoid incidents. In many cases rehabilitation professionals were themselves expected to draw on the workplace education that they received in crisis intervention [22] to figure out how to avoid or de-escalate violence that often was recurring in the context of the direct care they provided. For example, one participant described a patient who had a BSA because he has been known to yell completely unprovoked profanities at professionals, volunteers, and visitors:

I could be walking by him, and he could just sit there and swear at me for no reason. Like I hadn’t seen him all day and all of a sudden, he sees me, and he starts swearing at me. But then three hours later I walk by him again and he’s smiling at me. So, yeah, it’s just that – something might have irked him, but he’s not really able to tell us what irked him, to provoke that behaviour. (105, Complex Continuing Care)

The BSA for this patient also included that he could try to touch, kick or spit on people as well. When asked what she does in response to his behavior, she explained that because it was considered to be unavoidable, there were no specific strategies noted on his care plan for how to prevent or manage it, and thus ignoring him was the most common response:

We just ignore it. If he physically starts like charging at us, then we might get security, or another staff involved to try and calm him. But as long and he’s not doing any [physical] harm [because he is not within arms’ reach of anyone] then we just ignore him. Because there’s no reprimanding him. (105, Complex Continuing Care)

When asked what other strategies were available for the prevention of verbal or physical forms of patient violence, she, as well as other rehabilitation professionals familiar with the patient shared the following: limit setting; verbal de-escalation; diversion/distraction; use of “as needed” psychotropic medication; evasion techniques (e.g. walking around or moving out of reach of a seated patient if they try to grab or kick, moving a patient to the back of the room so they won’t be able to reach anyone to grab or kick); containment techniques (e.g. getting out of a grab, holding a patient); having a security guard follow a patient around or patrol the unit; and removing the patient completely from the rehabilitation program. These are all examples of strategies that participants noted they learned from violence prevention training, which included mandatory online modules for all staff as well as additional specialized training that was available to professionals in high-risk units (e.g., inpatient brain injury rehabilitation; specialized dementia unit). Such curricula typically covered common cues of aggression across the escalation continuum (e.g., raised voice, angry expression on face), de-escalation through interpersonal relations/verbal communication and behavioral management techniques (e.g., moving the patient to a quiet room, re-approaching for care at a later time), and personal physical safety techniques (e.g., ensuring that access to an exit is not blocked when in a patient’s room). The use of these strategies for violence prevention was also explicitly mandated within the violence prevention policies of the rehabilitation hospital (e.g., policy on when to call for a code white, which is an emergency warning code created to respond to a violent person). Thus, regardless of whether specific triggers or strategies were listed in a patient’s BSA, the use of this technology for violence prevention was ultimately perceived to be ineffective since rehabilitation professionals still had to rely on their clinical expertise and training to attempt to prevent incidents of violence from occurring.

#### Alarms

Another common technology that was available to all professionals was fixed emergency alarm systems, which included call buttons in patients’ rooms (wall-mounted or attached to bed) that could be used to alert the nursing station that help is needed. Some units also had desk telephones or mobile phones that staff could use to call for help via the nursing desk, security, or switchboard. These phones could also be used to announce a code white alert over the public address system (PA), which mobilized an emergency team of staff trained to manage violent situations from across the institution along with the security team. For the most part, participants reported that these technologies were ineffective as they were not always accessible and/or did not summon assistance fast enough. Describing her experience with the call buttons, one participant explained:

Sometimes you might be stuck in the washroom and it’s exactly on the other side where the [call button] is and you cannot get out from there, so then what are you supposed to do except yelling? Nothing. (104, Specialized Dementia Unit; Brain Injury)

Participants described how desk phones posed similar accessibility issues. The following is illustrative of this:

If … [an interaction with a patient] turned into a code white, like, I’d be walking away from [the patient] as [they’re] walking towards me, as I go [to] the phone and have this conversation. It makes no sense. And the phone’s over there, the door’s over here. Like, you know what I’m saying? ... [T]his is not a good situation. (101, Musculoskeletal).

Even if the phone could be reached, the same participant explained that they facilitate only indirect communication and create concerning delay in reaching help:

… getting to a phone, calling 555, having Switchboard listen to you and figure out what you’re doing, then going overhead, that process takes time … Because there is time wasted there, and in a code white situation…like, hold on a second, let me call 555. (101, Musculoskeletal)

In addition to these environmental accessibility challenges, the technology itself did not always work. The following participant describes how the mobile phone she was to carry with her to patients’ rooms was not working:

Sometimes you can [use this phone where you] just push the button and then it will go directly to the [nurses] station or even downstairs to the cop centre. But lately, we were not able to use those so you would actually have to go and find [another] phone, pick up the phone and call security or something like that, or just yell that I need help … [but] it’s over a month now that [these phones] are not working. (104, Specialized Dementia Unit; Brain Injury)

A personal security alarm (push button) worn as a pendant was also available to some of the participants. In addition to the convenience this afforded in terms of easy reach, it also has the benefit of directly alerting the security personnel about the need for help without having to announce a code white response over the PA system that would bring in multiple staff from across the hospital. As the following participant explains, the pendant button was perceived as being less likely to influence patients’ behavior, including eliciting more violence that can happen when calling a code white over the PA system:

[The pendant] can get some help in the moment, but you’re not causing mass panic and chaos … sometimes, you know, the amount of people that come running to a code white can escalate it more. You know, sometimes it’s more effective to de-escalate with a smaller group than with the whole world. (102, Complex Continuing Care).

However, despite these positive aspects of the personal alarms, some participants experienced them as heavy/cumbersome given all of the other things and devices they had to carry with them:

So, the button, like so it’s on you that you carry around you and you have access to activate from any location. And we’re all supposed to be wearing them. And I would say very often, people stop carrying them around, because they…weigh you down when you got your keys and your badge and everything. (109, Specialized Dementia Unit)

Whether these alarm technologies were used to summon general assistance from others on the unit or to mobilize an emergency response, these were not perceived as being effective for prevention since they were used once a violent incident was already occurring and/or professionals were not successful in preventing it; this was also explicitly stated in the code white policy that directed staff to use this code if the patient did not respond to verbal de-escalation techniques. Moreover, some participants were also reluctant to use these technologies because they perceived them as having the potential to impair care relationships with patients, including rapport and future interactions:

I mean if you call a code white you pretty much, you’re calling a code white because you need to because the person is trying to harm you or somebody else … And so, there’s always the potential that you’re going to have a hard time recreating the rapport you had before that happened. (103, Brain Injury)

#### Cameras

Another common form of technology that was available was closed-circuit television (CCTV) cameras that were located in public areas (e.g.,  unit hallways, gym). In most units, the video captured with these cameras was streamed to monitors located off the unit at the security desk. In some inpatient units it was streamed to monitors located at the nursing desk. However, these too were perceived as ineffective for violence prevention. A participant explains:

Would a camera be [effective] for violence prevention? I don’t think so unless a patient knows. For example, if someone is planning to be violent and they know there is a camera pointing right at them, maybe. Maybe if the patient was aware, it could prevent. But for the most part, I think it’s more … used in the aftermath of something going wrong, and that they would go back and look at cameras. (101, Musculoskeletal)

Even the use of the cameras for retrospective analysis was rare as it was challenging to obtain access to this footage. For example, when asked if she watched the footage from the CCTV cameras, one participant explained:

We don’t usually get to watch it … the security guard has to get special permission to have the footage released to them. And then we also have to be really specific about where and what time and what day the incident happened so that the security guard doesn’t have to review like a day’s worth of footage. (103, Brain Injury)

However, on locked inpatient units where the video footage was streamed to the nursing station in real-time, the use of this technology was seen as valuable for surveillance of patients who were known to be at risk for being physically aggressive to enable faster response time if an incident was caught on camera early enough. In this way, the technology was used more as a behavioral management strategy than for violence prevention. This is explained by a participant:

I don’t know about prevention, maybe … if someone was escalating … then you could kind of use the cameras to, like follow someone around and monitor them without having to follow, like physically follow them around. Because sometimes when ...someone’s, you know, aggressive or very frustrated, but you can set them off by being really close to them or following them if they see you. So, sometimes having the cameras allows you to kind of still maintain that safety but give them sort of the space that they need. (103, Brain Injury).

A noted challenge to the use of cameras in this way was the lack of dedicated staff for monitoring cameras in real time and thus professionals would only occasionally glance at the monitors while engaged in other tasks at the nursing station (e.g., doing chart reviews, documentation). Some participants also noted that this was ineffective since they did not know what to look for on the video feed in terms of less obvious visual cues or signs of escalation that an incident is imminent. The following participant describes this in terms of training that is needed:

But how to know when to intervene…when you monitor… a good comparison is cardiology… I worked full-time in psychiatry [before coming to this unit], but as a part-timer I worked in intensive care, in surgery, in the private hospital. Anyway, so, I know that if you monitor [for signs of a cardiac episode], you don’t need to wait. You have the facts. You can catch it before [it happens] if you monitor well. But to monitor [for violence] well, you have to educate people what to look for. (106, Brain Injury)

Moreover, even if professionals were able to use the cameras in this way, they would still have to prevent it on their own using de-escalation and crisis intervention techniques, which were not always considered to be effective:

So, they’re about to become agitated, so what? So, what am I going to do differently? And I mean, I think I don’t know. Like I think we can start to see those early signs of them starting to become aggressive and I think the problem is we don’t always know what to do with that or how to change our approach to prevent that. Like with the patient I know who was, you know, turning purple and flailing their arms…like, I wouldn’t need, you know, a camera or bed sensor or anything, or heart rate monitor to tell me that. I can easily, like plain as day, see that, but it’s figuring out what works for this patient to de-escalate them and how, what I can do to do that. If the machines could de-escalate for us, that would be really useful [chuckle]. (102, Complex Continuing Care).

## Discussion

We found that rehabilitaion professionals perceived the technogies that were implemented in their institution to prevent incidents of violence (e.g., patient-patient and patient-staff) as ineffective due to limitations in their design, common malfunctioning, limited organizational resources, and incompatibility with the culture of care. Our study contributes to addressing a gap in knowledge by being the first to explore the perceptions and experiences of rehabilitation professionals regarding how technologies are used (or not) for violence prevention, and their perceptions regarding their efficacy and impact. To date, most research on the prevention of violence in healthcare has focused on quantifying the scope of this public health problem and characterizing perpetrators and victims, and there is limited evidence on the effectiveness of commonly recommended interventions [2, 23, 24]. Moreover, the existing research has primarily focused on nurses and other healthcare professionals in emergency rooms and psychiatric hospitals, leaving a paucity of research on other types of professionals or violence prevention interventions in rehabilitation settings.

Identifying and flagging patients who had previously been violent to alert professionals of this potential threat is a standardized component of organizational violence prevention programs across jurisdictions [2, 6, 9]. While there is some evidence that such practices are valued by professionals and may facilitate their use of preventative measures [1, 7, 23], in our study, participants reported that the electronic flagging system was not useful as a visual cue or for recognition of risk because of its lack of prominence and visibility in the electronic patient health record. This suggests that a more active flagging system, where staff need to acknowledge having read/seen the flag in order for their work flow to continue, may be more effective [9]. However, in our case, increasing the visibility of flags may not enhance effectiveness as participants also indicated that another reason these were ineffective was that they were already aware of patients who have been documented as being violent using other means (e.g., familiarity from working with the patient, co-workers sharing this information, admission notes). Instead, a more effective flagging system would be one that is not static, general, and permanent, but rather one that signals the level and nature of the risk to staff [5, 9]. While there is some suggestion in the literature [7, 9] that even a passive and static electronic flagging system may be effective in reassuring staff because it enables them to plan pre-emptive measures, our participants did not describe such reassurance. The absence of reassurance may be due to what Patterson and colleagues [5] have identified as “alert fatigue” that can result when an alert has been in place for a long time and professionals become less vigilant to it. Providing ongoing training to remind professionals of the importance of using the flagging system alongside ongoing re-evaluation and updating flags with additional information from new incidents and/or new strategies implemented could help to mitigate this.

Another common example of a technology that is widely advocated for violence prevention in the literature [2, 23] but that participants in our study perceived as not working as intended and thus ineffective, were emergency alarm systems. For example, participants described that these technologies could fail, and even when they worked, they did not summon assistance fast enough or were inaccessible in terms of reach. Even when participants had access to a personal alarm that was worn around their neck, they were reluctant to use them because they were cumbersome given all the other items that they carried with them. Research on the use of emergency alarms and other technologies in healthcare (e.g. vitals monitors, sensors, telehealth) has similarly identified examples of poor design and malfunctioning, with some suggestions that this may lead to mistrust that restricts their use [1, 25–28]. For our participants, there was not this explicit connection between such failures and mistrust, however, there was the suggestion that malfunctioning did negatively influence the perceived effectiveness of the alarms. Given the lack of studies that have specifically explored the effectiveness of alarms in reducing violence in healthcare [24] it is unclear how effective they would be, even in the absence of these failures.

We also found that participants reported that the use of closed-circuit cameras for remote surveillance of patients for violence in public areas was ineffective as a violence prevention strategy, even in units where the video was streamed in real time to the nursing station. This was in part attributed to the lack of dedicated time/staff to monitor patients as well as a lack of training in terms of what to look for and anticipate regarding a violent event. There is some research that suggests that the use of remote video monitoring can be effective for prevention of violence [8, 29] but only when all patient areas are monitored, and when there are dedicated and trained video monitoring technicians who can respond to incidents in real-time using a two-audio communication system that allows for both remote and on-site response to an incident. This research also suggests that video cameras may be most effective with patients who have a documented history of being at risk of being violent and have known triggers/cues. Yet, as reported by Quigley and colleagues [8] in a study of patient engaged electronic video monitoring, 85% of patients who had exhibited violence during the time of the study were not being monitored as they had not been identified as being at risk at its start. This may reflect the broader issue of professionals underreporting incidents of violence and a reluctance to identify violent patients [5, 23, 30], which has been attributed to a number of factors including concern that reporting/labelling stigmatizes patients, and that professionals normalize violence as part of their work [1, 7, 20].

Normalization of violence as part of the job of healthcare could be linked to a culture of care that fosters the expectation that professionals manage violence on a daily basis; this is communicated by organizational policies and training on prevention that emphasize the responsibility of workers to identify and manage violence through de-escalation and other client handling practices [1, 20, 28, 31]. Our participants similarly described this normalization of violence in terms of the training they received in non-violent crisis intervention and self-defence strategies. Such strategies are considered the first-line intervention for imminent violence as documented in professional guidelines and in research and policy for violence prevention in healthcare [32, 33]. While these strategies are less harmful to patients than restrictive measures (e.g., restraint and seclusion), they make professionals more vulnerable and hold them primarily responsible for violence prevention. This is despite the limited evidence there is on the effectiveness of de-escalation strategies, including which of these are most effective and under what conditions [32]. Further, the use of these strategies is also based on the assumption that the cause of violence is found in individual patients, which leaves entirely unaddressed the underlying systemic and structural causes of violence [1, 19–21, 34].

The use of flagging, alarms, and cameras as violence prevention strategies is premised on the same reductionist logic by focusing prevention on the identification and surveillance of individual “violent patients.” Yet until and unless the structural causes of violence are considered in remediation strategies, technology will always fall short of being an effective prevention strategy. For example, there is ample research that has linked heavy workloads, low and inappropriate mix of staffing levels, and long wait-times with violence between patients and towards professionals [1, 19, 21, 35]. Further, the healthcare environment, including inappropriate temperatures, confined spaces, excessive noise, overcrowding, patients’ lack of privacy, noise pollution, and aesthetic deprivation have also been identified as contributing factors to aggression [34, 36–38]. It is our argument that prevention efforts, including the development and implementation of new technologies, should thus be reoriented to address these and other structural factors. Indeed, others have similarly argued that implementation of technologies for violence prevention should be anchored in a broader organizational commitment to a safer workplace, including enhancements to the physical environment and their ongoing review, and modification and enforcement of implemented protective measures [7, 9, 28].

### Limitations

Given that our study is the first to explore the use of technologies for violence prevention in a rehabilitation setting, it will be important to further explore this topic in other similar settings to determine the transferability of our findings. Moreover, there was the suggestion that the use of these technologies may negatively impact patients and the care professional/patient relationship, however, because this wasn’t an explicit focus of our study, we are unable to determine how salient of an issue this is. There is some research that suggests that the use of technologies in non-rehabilitation settings may have this negative impact, including concern that these technologies may stigmatize patients and that noncompliance with their use may be a function of the desire to protect patients from this [9]. Therefore, it will be important to explore these phenomena in greater depth in other rehabilitation settings and with a larger number of diverse participants to gain additional insights and to explore differences and similarities among different subgroups of professionals (e.g. based on gender, years of work, cultural norms, type of profession). Finally, while the technologies we explored were implemented alongside broader organizational initiatives for violence prevention, and our participants did speak to policy and training regarding violence prevention, our focus was the technology per se and thus we did not explore in any comprehensive way the interrelationship between these and other dimensions of violence prevention initiatives in this setting. We therefore are unable to address the ways in which these broader organizational initiatives might support or constrain professionals’ understanding and use of technologies for violence prevention. Ethnographic research would be well-equipped to further our understanding of this complexity and is thus an important next step in exploring how these and other technologies are used (or not), and their efficacy and impact [39].

## Conclusions

Our qualitative study demonstrates that rehabilitation professionals perceive technologies as largely ineffective for violence prevention due to poor design features, common malfunctioning, limited resources, and incompatibility with the culture of care. Given that the development and implementation of technology is increasingly advocated for enhancing efforts to prevent violence in healthcare settings, our findings offer important direction for future research in this area.

## Data Availability

The data is not openly available in order to protect the participants’ privacy and confidentiality, particularly given the small sample size and the study’s setting specificity but are available from the corresponding author on reasonable request.
